# Predictive markers for efficiency of the amino-acid deprivation therapies in cancer

**DOI:** 10.3389/fmed.2022.1035356

**Published:** 2022-11-03

**Authors:** Vadim S. Pokrovsky, Louay Abo Qoura, Elena Morozova, Victoria I. Bunik

**Affiliations:** ^1^Laboratory of Experimental Oncology, Research Institute of Molecular and Cellular Medicine, People’s Friendship University of Russia (RUDN University), Moscow, Russia; ^2^Laboratory of Combined Treatment, N.N. Blokhin National Medical Research Center of Oncology of Ministry of Health of Russian Federation, Moscow, Russia; ^3^Department of Biotechnology, Sirius University of Science and Technology, Sochi, Russia; ^4^Engelhardt Institute of Molecular Biology of the Russian Academy of Sciences, Moscow, Russia; ^5^A.N. Belozersky Institute of Physicochemical Biology, M.V. Lomonosov Moscow State University, Moscow, Russia; ^6^Faculty of Bioengineering and Bioinformatics, M.V. Lomonosov Moscow State University, Moscow, Russia; ^7^Department of Biological Chemistry, Sechenov First Moscow State Medical University, Moscow, Russia

**Keywords:** amino acid deprivation therapy, L-asparaginase, arginine deiminase, arginase, methionine γ-lyase, L-lysine oxidase, asparagine synthetase, methionine synthase

## Abstract

Amino acid deprivation therapy (AADT) is a promising strategy for developing novel anticancer treatments, based on variations in metabolism of healthy and malignant cells. L-asparaginase was the first amino acid-degrading enzyme that received FDA approval for the treatment of acute lymphoblastic leukemia (ALL). Arginase and arginine deiminase were effective in clinical trials for the treatment of metastatic melanomas and hepatocellular carcinomas. Essential dependence of certain cancer cells on methionine explains the anticancer efficacy of methionine-g-lyase. Along with significant progress in identification of metabolic vulnerabilities of cancer cells, new amino acid-cleaving enzymes appear as promising agents for cancer treatment: lysine oxidase, tyrosine phenol-lyase, cysteinase, and phenylalanine ammonia-lyase. However, sensitivity of specific cancer cell types to these enzymes differs. Hence, search for prognostic and predictive markers for AADT and introduction of the markers into clinical practice are of great importance for translational medicine. As specific metabolic pathways in cancer cells are determined by the enzyme expression, some of these enzymes may define the sensitivity to AADT. This review considers the known predictors for efficiency of AADT, emphasizing the importance of knowledge on cancer-specific amino acid significance for such predictions.

## Introduction

More than 50 years of research on cancer cell metabolism has concluded that a deficiency of certain amino acids inhibits the growth and proliferation of tumor cells much more than that of normal cells (1). Hence, àmino-acid degrading enzymes have been studied for their potential to treat cancer since 1960s, starting with a report of Broome et al. on antilymphoma effects of L-asparaginase (2, 3). Metabolic reprogramming commonly occurs in tumor cells to sustain the high nutritional requirements for carcinogenesis and growth (4). Many cancer cells develop an auxotrophic response to certain amino acids, such as methionine, arginine, and asparagine (5–7). For the treatment of acute lymphoblastic leukemia (ALL), bacterial L-asparaginase has been approved since 1970s (8). Arginine-depleting enzymes are suggested to treat metastatic melanoma (9). Arginine deiminase and recombinant human arginase 1 that deplete serum arginine, have recently been evaluated in phase II clinical trials (10, 11). Summarizing available information on the molecular mechanisms of the cancer-specific action of these amino-acid-degrading enzymes, our review draws attention to the amino-acid-replenishing counterparts among potential predictors of the efficacy of anticancer action of the amino acid-degrading enzymes.

## L-asparagine depletion therapy

**FIGURE 1:**
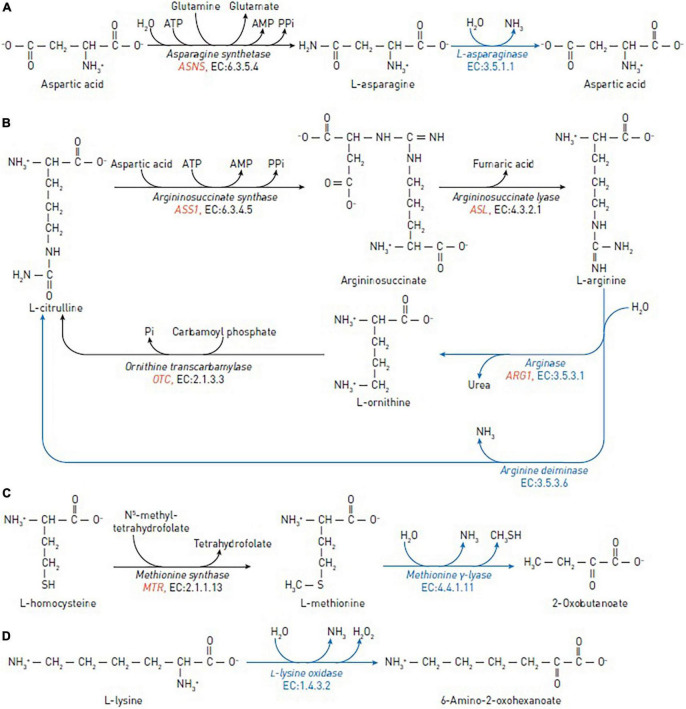
Reactions catalyzed by the amino-acid degrading enzymes used in cancer therapy (blue arrows) and reactions catalyzed by human enzymes that may affect the efficacy of the anticancer enzymes (black arrows). **(A)** The reactions of asparagine synthetase and asparaginase in asparagine depletion therapy. **(B)** The reactions of argininosuccinate synthase, argininosuccinate lyase, ornithine transcarbamylase, arginine deiminase and arginase in arginine depletion therapy. **(C)** The reactions of methionine synthase and methionine γ-lyase in methionine depletion therapy. **(D)** The reaction of L-lysine oxidase in lysine depletion therapy. The human genes encoding enzymes discussed as biomarkers are in red italic.

In normal cells, L-asparagine is produced by asparagine synthetase (EC 6.3.5.4), which catalyzes the synthesis of asparagine from aspartate, using glutamine as a nitrogen source (Figure 1A). Leukemic and certain other types of cancer cells are dependent on exogenous sources of asparagine due to low expression of the asparagine-synthetase-encoding gene *ASNS* (12, 13).

During the last 50 years, bacterial asparaginase have been widely used for acute lymphoblastic leukemia treatment (8). This enzyme has recently received attention in the treatment of advanced extra-nodal NK/T-cell lymphoma (14, 15). *In vitro*, it is shown that asparaginase-resistant cells become more sensitive to asparagine depletion when *ASNS* expression is downregulated, as observed in lymphoblasts (K562) and non-Hodgkin’s lymphoma (Karpas299) cells (16, 17). Immunohistochemistry of ASNS protein is available to assess the enzyme expression (18), in addition to estimation of mRNA levels. Tissue microarrays have been utilized to identify asparagine-synthetase-low cancer cells within a number of solid cancer subtypes in non-hematological malignancies (19). Based on this study, asparagine synthetase level in the tumor cells is proposed as a predictive biomarker for their sensitivity to asparaginase therapy (19). In 2006, FDA approved the first-line treatment of patients with ALL by chemically modified form of *E. coli* asparaginase (Oncaspar^®^). The modification by polyethylene glycol (i.e., pegylation) extends half-life of asparaginase *in vivo* (20). Erythrocytes-encapsulated asparaginase (GRASPA^®^, Erytech) represents another innovative formulation, that has been investigated for treatment of solid tumors, including pancreatic cancer (21). Conjugation with heparin-binding peptides or directed mutagenesis are also employed for the therapeutic usage of asparaginase isolated from bacterial sources (22–25).

A wide range of *ASNS* expression in different tissues is reported. Particularly high levels of expression are detected in the brain, testes, thyroid, and normal exocrine pancreatic cells. Acute lymphoblastic leukemia and hepatocytes typically have low *ASNS* expression (26, 27), and more than 50% of pancreatic ductal adenocarcinomas have very low *ASNS* expression (28). Thus, asparaginase may be suggested as effective drug for the treatment of pancreatic ductal adenocarcinomas lacking *ASNS* expression. Limited asparaginase efficiency in fighting many solid cancers, such as prostate and ovarian cancer, is believed to be due to the medium/high expression of *ASNS* in these cancer types (29, 30). Moreover, it has been shown that *ASNS* hypermethylation results in low *ASNS* protein expression in liver and gastric cancer cells, making them more susceptible to asparaginase therapy *in vitro* and *in vivo* (31). However, many studies have demonstrated that acute lymphoblastic leukemia can still be inhibited by asparaginase even when *ASNS* is expressed (32, 33). Additionally, no association was found between the asparaginase sensitivity and various levels of asparagine synthetase in acute myeloid leukemia subgroups in human cancer cells, probably explained by post-translational control of asparagine synthetase (34, 35). Asparagine auxotrophy not only has the apparent implication in heightened sensitivity to asparagine depletion and, therefore, to asparaginase therapy, but also implies the cancer cells’ tight reliance on external supplies of the amino acid even under normal growth conditions (26).

In summary, *ASNS* expression is suggested as a marker for clinical prediction of asparaginase resistance (19), supported by majority of the studies revealing a strong negative correlations of the asparaginase efficacy with the *ASNS* gene expression. High *ASNS* expression may contribute to asparaginase resistance of tumor cells. Low *ASNS* expression in tumor cells, including certain solid cancers, such as pancreatic ductal adenocarcinomas, could be used to suggest patients the treatment with asparaginase-added chemotherapy.

## L-arginine depletion therapy

L-arginine is a crucial semi-essential amino acid involved in a variety of physiological functions, including cellular proliferation, through the arginine-dependent signaling pathways. These pathways involve the arginine-dependent generation of nitric oxide and polyamines, as well as activation of mTOR, a nutrient-sensing kinase strongly implicated in tumorigenesis. Arginine is synthesized from citrulline in two steps: (1) Argininosuccinate synthase converts L-citrulline and aspartic acid to argininosuccinate; (2) argininosuccinate lyase converts argininosuccinate to arginine and fumaric acid (Figure 1B).

Arginine deiminase (EC 3.5.3.6) and arginase (EC 3.5.3.1) degrade arginine. Arginine deiminase is widely distributed in bacterial organisms and certain anaerobic eukaryotes, and is isolated from a variety of sources, such as *Pseudomonas putida*, *Giardia intestinalis*, *Streptococcus pyogenes*, *Mycoplasma* spp. (36–39) and others. Arginine deiminase catalyzes the irreversible conversion of arginine to L-citrulline and ammonia (40). This process produces anti-tumor effect in a wide range of human cancers, including hepatoma, malignant melanoma, malignant fibrosarcoma, squamous cell carcinoma, nasopharyngeal carcinoma, and lung carcinoma *in vitro* and *in vivo* (41, 42). Arginine deiminase has been effective in phase II clinical trials for metastatic melanoma, hepatocellular carcinoma, and malignant mesothelioma (42–46). Therapeutic usage of pegylated arginine deiminase, possessing antiproliferative action against human leukemia cells (47).

Arginase is a manganese-dependent enzyme catalyzing the arginine conversion to ornithine and urea. Recombinant human arginase 1 has previously been shown *in vitro* to suppress non-Hodgkin’s lymphoma cells (48), prostate cancer cells (LNCaP, DU-145, and PC-3) (49), melanoma cells, laryngeal squamous cell carcinoma (50), leukemia cells (51), non-small cell lung cancer (NSCLC) (52), and ovarian cancer cells (53).

Arginine-depriving enzymes, such as arginase and arginine deiminase, may be useful to fight cancer cells (54) which lack significant levels of the arginine-replenishing enzymes: the *ASS*-encoded argininosuccinate synthase and *ASL*-encoded argininosuccinate lyase. On the other hand, overexpression of arginase in cells significantly increases the concentration of L-ornithine, that is recycled to arginine by ornithine transcarbamylase/argininosuccinate synthase (Table 1). These arginine re-synthesizing reactions provide resistance to the arginase treatment in several malignancies (5).

**TABLE 1:** Enzyme expression as predictive markers for treatment cancer with amino-acid degrading enzymes.

Therapeutic enzyme	Amino acid degraded	Human gene(s) for enzyme(s)-predictor(s)	Pathway of enzyme-predictor
L-Asparaginase	L-asparagine	ASNS*	Asparagine biosynthesis
Arginine deiminase and arginase	L-arginine	ASS*/ASL*/ OTC***	Arginine biosynthesis from citrulline or regeneration from ornithine
Methionine γ-lyase	L-methionine	MTR**	Methionine regeneration from homocysteine
L-Lysine oxidase	L-lysine	DHTKD1***/ GCDH***/ SIRT5***	Protein glutarylation***

*Validated in clinical trials.

**Validated in non-clinical trials.

***Putative predictors/pathways, based on metabolism and/or biological functions of L-arginine and L-lysine.

Deficiencies of argininosuccinate synthase and/or *OTC*-encoded ornithine transcarbamylase are regarded to be prognostic biomarkers and predictors of sensitivity to the arginine deprivation (55). Human melanoma (56), hepatocellular carcinoma (57), colon cancer (HT29) (58) and prostate carcinoma (59) have been demonstrated to be sensitive to arginine depletion by arginine deiminase due to low or negligible expression of *ASS/ASL* genes *in vitro* and *in vivo* (60–62). In contrast, arginine deiminase is ineffective in the treatment of cancers with high or medium expression of *ASS*, like ovarian (63), and colon cancer *in vitro* and *in vivo* (SW480 and HCT116) (58).

More than a 75% reduction of argininosuccinate synthase activity in cancer cells, compared to their healthy counterparts, is a positive prognostic marker for arginine deiminase efficacy (46). Expression of *ASL* and *OTC* genes may also provide valuable information to predict the efficacy of arginine-depletion therapy (52, 64).

## L-methionine depletion therapy

L-methionine is an essential amino acid that contains a sulfur atom and participates in such a crucial function as DNA methylation. In normal cells, methionine can be recycled by re-methylation of homocysteine, catalyzed by the cobalamin-dependent enzyme methionine synthase (Figure 1C) or by betaine-homocysteine methyltransferase in the liver (65). Experiments show that many cancer cells, including leukemia (L1210 and J111) (66), breast (MDA-MB231, MCF7, SKBR3, and T47D) (67), lung (A2182 and SK-LU), kidney (A498), CNS (SK-N-SH), prostate (PC-3), and colon (SK-CO-1 and loVo) (68) cancer cells, cannot proliferate when methionine in growth medium is replaced with homocysteine *in vitro*.

Methionine γ-lyase (EC 4.4.1.11) is a bacterial pyridoxal-5’-phosphate-dependent enzyme which catalyzes γ-elimination of L-methionine to generate α-ketobutyric acid, methyl mercaptan and ammonia (69). The enzyme has been isolated from *Pseudomonas putida* (70), *Trichomonas vaginalis* (71), *Clostridium sporogenes* (72), *Entamoeba histolytica* (73), *Citrobacter freundii* (74), *Clostridium tetani* (75), and others. Methionine γ-lyase from *Pseudomonas putida* inhibited the growth of neuroblastoma (LAN-1 and NMB-7) (76), Yoshida sarcoma and lung cancer (H460) (77), advanced breast cancer (78, 79), renal cancer and lymphoma (78), human colon cancer xenografts (HCT15, HT29, COLO205, and SW620) (80) and glioblastoma (81). Pegylation of methionine γ-lyase was used to increase serum half-life and reduce immunological reactions *in vivo* (82). No clinical toxicity was found after treatment with methionine γ-lyase in a pilot phase I trial on human cancer patients (83).

Compared to normal tissues, cancer cells have a higher requirement for methionine synthase activity and may thus be more sensitive to methionine synthase inhibition (84–87). Large number of tumor cell lines, including melanoma, glioblastoma, colon, lung, breast, bladder, and kidney tumors, lack the normal pathway of methionine re-synthesis (88, 89). Methionine synthase is encoded by *MTR* gene whose polymorphism may affect DNA methylation and thus contribute to cancer development (90). Furthermore, the A2756G (rs1805087) substitution in the *MTR* gene plays a role in the progression of breast and prostate cancer via the pathway of the methyl group transfer, which is involved in both DNA methylation and DNA synthesis (91, 92). Ile22Met mutation (A66G) of the *MTRR-*encoded methionine synthase reductase is linked to folate, vitamin B_6_, or vitamin B_12_ levels in colorectal cancer. However, no statistically significant correlation between the Ile22Met mutant and risk of pancreatic cancer is reported (93). Instead, pancreatic cancer risk is influenced by His595Tyr mutation of methionine synthase reductase (94). *MTRR* gene suppression may be effective in the treatment of pancreatic ductal adenocarcinomas (94).

Thus, analysis of available data suggests that expression of methionine synthase could predict sensitivity of cancer cells to methionine-cleaving enzymes. Cells with low expression of methionine synthase are expected to be more sensitive to treatment with methionine γ-lyase. However, further studies are needed to establish the diagnostic significance.

## Lysine depletion therapy

L-lysine is an essential and abundant amino acid in humans. In addition to proteinogenesis (95), L-lysine may be used for ketogenesis. An important energy source under starvation (96, 97), ketogenesis is also involved in responses of cancer cells to therapeutic agents (98). Besides, L-lysine catabolism through the *DHTKD1*-encoded 2-oxoadipate dehydrogenase produces glutaryl-CoA for protein glutarylation (97, 99, 100). Particularly glutarylation of histones, associated with gene activation (99), and regulation of pyruvate dehydrogenase by glutarylation (100) may be involved in metabolic transformation of cancer cells, causing their specific sensitivity to L-lysine depletion. L-lysine α-oxidases (EC 1.4.3.14) catalyze the oxidative deamination of L-lysine, resulting in the production of α-keto-ε-aminocaproate, ammonia, and H_2_O_2_ (Figure 1D). Over the last 40 years, several biological effects of L-lysine α-oxidases have been described, including antiviral, antimicrobial, anti-protozoa, anti-metastatic, and antitumor (101–105).

L-Lysine α-oxidase from *Trichoderma cf. aureoviride Rifai* has significant cytotoxicity against the following human cancer cell lines: K562, LS174T, HT29, SCOV3, PC3, and MCF7 *in vitro* (102) and PC12 (106). Human colon cancer xenografts HCT116 and LS174T, as well as breast adenocarcinoma T47D, demonstrated high sensitivity to L-lysine α-oxidase (102). *Ophiophagus hannah* venom-derived L-lysine α-oxidase inhibited the growth and proliferation of PC-3 prostate cancer xenografts (107). Depending on the dosage, the enzyme from *Agkistrodon acutus* suppressed the development of hepatoma 22, sarcoma 180, and Ehrlich carcinoma (108). In many species, L-lysine α-oxidase from *Trichoderma harzianum Rifai* and *Tr. viride Y244-2* reduced malignant properties of solid tumors (105, 109). The most susceptible murine transplantable tumors were melanoma B16, breast adenocarcinoma Ca755, ascitic hepatoma 22, cervical cancer RSHM5, and colon carcinoma AKATOL (110). It may be hypothesized that the susceptibility is linked to the L-lysine-dependent induction of specific metabolism of these cancer cells by protein glutarylation. In this case, expression of the enzymes determining the levels of glutaryl-CoA, i.e., the proteins encoded by the *DHTKD1* and *GCDH* genes, and protein deglutarylation, i.e., *SIRT5* protein, may comprise the markers of efficiency of the L-lysine-depleting therapies.

## Conclusion

Recent discoveries of the molecular pathways of amino acid metabolism and their regulation in tumor cells highlight specific features of tumor metabolism that may be used for prediction of efficacies of therapeutic strategies based on depletion of amino acids. An increased need for amino acids caused by rapid proliferation of cancer cells, contributes to metabolic abnormalities of these cells. In comparison to traditional anticancer treatments, those involving the amino-acid-degrading enzymes offer several advantages: (1) potent effects on specific amino acids indispensable for cancer cells; (2) low toxicity; (3) usage for combinatorial therapies; (4) existence of biochemical markers to predict the treatment responses. The predictors are expression of genes, such as *ASNS* for asparagine depletion therapy; *ASL*, *ASS*, and *OTC* for arginine depletion therapy; *MTR* for methionine depletion therapy. More clinical research is necessary to extend the list of such biomarkers and assess their prognostic value demonstrated in pilot studies.

## Author contributions

VP, LQ, and EM wrote the manuscript. VB reviewed and edited the manuscript. All authors contributed to the article and approved the submitted version.
